# Production Response and Digestive Enzymatic Activity of the Pacific White Shrimp *Litopenaeus vannamei* (Boone, 1931) Intensively Pregrown in Microbial Heterotrophic and Autotrophic-Based Systems

**DOI:** 10.1100/2012/723654

**Published:** 2012-05-02

**Authors:** Manuel J. Becerra-Dórame, Marcel Martínez-Porchas, Luis R. Martínez-Córdova, Martha E. Rivas-Vega, José A. Lopez-Elias, Marco A. Porchas-Cornejo

**Affiliations:** ^1^Departamento de Investigaciones Científicas y Tecnológicas de la Universidad de Sonora, Boulevard. L.D. Colosio s/n, entre Reforma y Sahuaripa, Edificio 7G, Hermosillo, Sonora, 83000, Mexico; ^2^Centro de Investigación en Alimentación y Desarrollo A.C. Carretera a La Victoria Km. 0.6, Hermosillo, Sonora, 83304, Mexico; ^3^Centro de Estudios Superiores del Estado de Sonora, Carretera a Huatabampo y Periférico Sur, Navojoa, Sonora, 85870, Mexico; ^4^Centro de Investigaciones Biológicas del Noroeste, Guaymas, 85454 Sonora, Mexico

## Abstract

Shrimp postlarvae were reared into different microcosm systems without water exchange; a traditional system based on simple fertilization to improve microalgae concentration (control), an autotrophic system (AS) based on the promotion of biofloc and biofilm by the addition of fertilizer and artificial substrates and a heterotrophic system (HS) based on the promotion of heterotrophic bacteria by the addition of nitrogenous and carbonaceous sources and artificial substrates. Better growth performance and survival were registered in shrimp from the AS and HS compared to the control. Feed conversion ratios were below 0.7 for all treatments, but AS and HS were significantly lower than the control. Regarding digestive performance, no significant differences were observed for trypsin, amylase and lipase activities among AS and control shrimp; however, shrimp from HS showed a higher trypsin and amylase activities, suggesting a higher digestive activity caused by the presence of microbial bioflocs. The presence of biofilm and bioflocs composed by either autotrophic or heterotrophic organisms in combination with formulated feed improved the growth performance and survival of shrimp. Apparently, such combination fits the nutritional requirements of shrimp.

## 1. Introduction

Feed and feeding may represent up to 50% of the operative costs in shrimp aquaculture [1]; these costs could even be higher depending upon the intensity of the culture system. Additionally, unconsumed feed is the main potential source of deterioration on the water quality, impacting the effluent-receiving ecosystems [2]. In order to advance toward the sustainability of aquaculture, it is absolutely necessary to optimize not only the feed formulations but also the feeding practices. One of the most interesting and promising alternatives in this context is the promotion and use of natural feed, which has proven to provide a high proportion of the nutrients required for farmed shrimp, especially in the semi-intensive systems [3–5]. 

Up today, organisms from zooplankton communities have been the most used as natural feed for aquaculture purposes [6–8]. Microbiota has been used mostly as probiotic to improve the health status of the farmed organisms, or to maintain adequate environmental conditions within the culture units [9]. Traditionally, bacteria and other microorganisms have not been considered important in the feeding of farmed shrimp, probably because of their small size and biomass. However, their extremely great replication rate and nutritional composition make them an important source of food [10]. At present, diverse types of microorganisms are being used as direct feed for farmed shrimp at nursery, pregrown and growout phases [11, 12]. Since shrimp are unable to consume bacteria directly from the water column, natural or artificial substrates are usually introduced into the farming units to enhance the formation of biofilms or bioflocs, in which bacteria as well as other microorganisms such as microalgae, cyanobacteria, yeast, nematodes, protozoans, ciliates, and so forth, are important constituents forming aggregates. To promote the biofilm formation, fixed substrates made of nylon mesh or commercial Aquamats, are commonly used [13, 14]. For biofloc formation, floating substrates based on wheat bran, sugar cane bagasse, and other biomaterials are employed. For instance, the pink shrimp (*Farfantepenaeus paulensis*) has been nursed in a microbial suspended bioflocs based system, achieving better growth and feed conversion ratios (FCR) compared to the traditional system [11]. The authors found that the flocs were composed by detritus in the form of flocculated matter, colonized by heterotrophic bacteria, cocoid and filamentous cyanobacteria, flagellate and ciliate protozoans, and rotifers. Diverse studies have revealed that bioflocs and biofilms usually contain high-quality lipids and protein (PUFA and HUFA); additionally they contain high concentrations of vitamins [15, 16]. The microorganisms composing the bioflocs and biofilms may vary widely depending on diverse factors such as nutrient proportions, light intensity, and other environmental parameters [10]. The induction of those autotrophic and/or heterotrophic communities within aquaculture units can be achieved by manipulating the carbon/nitrogen ratio and light intensity [11, 12]. Both types of communities have been reported to have positive effects on the production response of the farmed shrimp. For instance, penaeid shrimp have been reared in Brazil, using heterotrophic communities (bioflocs and biofilms) at densities has high as 6,000 organisms*·*m^−3^, obtaining excellent growth and survival [11, 15, 17]. Regarding autotrophic communities, some shrimp farms in South America are beginning to use autotrophic systems, based mostly on microalgae (diatoms and flagellates), but contrarily to the traditional system, they introduce floating microsubstrates to enhance the production of bioflocs, and artificial fixed substrates to allow the formation of biofilms.

Apart from the positive effect of microbial communities associated to bioflocs and biofilms on the production response of the farmed organisms, recent evidence suggest that the nutritional status of shrimp is improved, whereas immunological condition is not affected [8, 14]. The nutritional condition of farmed organisms is consequence not only of the feed consumed during the culture but also of their digestive physiology. The digestive response is influenced by many extrinsic and intrinsic factors such as feed and metamorphic changes [18–20].

Based on the above information, the objective of the present study was to evaluate the effect of autotrophic and heterotrophic microbial based systems, on the productive response and digestive physiology of the Pacific white shrimp *L. vannamei* intensively farmed at pregrowout phase.

## 2. Materials and Methods

The study was done during nine weeks in the facilities of CIBNOR at Guaymas, Sonora, Mexico. A single-factor experimental design with three replicates per treatment was performed. The treatments consisted of: a heterotrophic-based system (HS), an autotrophic-based system (AS), and a control (C) equivalent to the traditional system. The experimental units were plastic tanks (300 L) provided with constant aeration from a 1 hp blower, through plastic tubing and air diffusers. Three tanks per treatment were employed. The tanks for the AS and control were exposed continuously to sunlight. The units for HS were put under constant shadow and covered with a black mesh layer to minimize the sunlight exposure. The units of treatments AS and HS were provided with artificial substrates (plastic mesh; 1 m^2^ per tank) and with wheat brand (20 g per tank*·*week^−1^) to enhance the formation and proliferation of biofilms and bioflocs, respectively. In the tanks of the AS the proportion *C* : *N*  was maintained around 5 : 1 using an agricultural fertilizer (*N* : *P* = 40 : 4). In the units of HS, the *C* : *N* was maintained around 20 : 1, by using molasses to provide organic carbon, following the specifications of Avnimelech [16].

White shrimp postlarvae (PL-30; *Litopenaeus vannamei*) obtained from a commercial hatchery were stocked in the experimental units at a rate of 250 shrimp*·*m^−3^. No water exchange was done during the experiment, and only the evaporated water was replaced each three days with filtered, aerated, and dechlorinated freshwater. The organisms were additionally fed a commercial diet with 35% of crude protein at a rate of 8% of total biomass per day. 

Water quality variables such as pH, dissolved oxygen, chlorophyll-a, salinity, and temperature were daily recorded in the treatments, using a multiparameter sensor YSI 6600 (Yellow Springs, OH, USA). Microalgae and bacteria concentrations in the water column were calculated by performing the methods described by Ballester et al. [11]. The biological composition of the biofilm was examined at the last day, by scraping in a surface of 10 cm^2^ of the artificial substrates of each unit as suggested by Burford et al. [21].

Feed supplied was weekly adjusted by weighting 30 shrimp from each experimental unit. At the end of the trial, all shrimp were counted and weighed to obtain the final survival, final weight gain, specific growth rate, and FCR (feed provided/shrimp biomass gained). The specific growth rate (SGR) was calculated by the following equation:

(1) $$\text{SGR}=\left[\frac{\text{Ln}\left(\text{Final.weight}\right)-\text{Ln}\left(\text{Initial.weight}\right)}{T_{1}-T_{0}}\right]*\left(100\right).$$

Samples of 20 shrimp in molting stage C were collected from each treatment at the end of the trial to evaluate the digestive physiology in terms of trypsin-like activity, amylase activity, and lipase activity. Hepatopancreas homogenates were prepared. Samples from each treatment were homogenized in 3 volumes of distilled water. The homogenate was centrifuged at 11,300 ×g at 4°C for 20 min. The supernatant was used for the following determinations.

**Trypsin-Like Activity**

Benzoyl-Arg-p-nitroaniline (BAPNA) 0.1 mM was used as substrate. Ten microlitres of the enzymatic extract, 1.25 mL of Tris buffer 50 mM pH 7.5, containing 20 mM CaCl_2_ and 50 *μ*L of the substrate were mixed. After 10 min, the enzymatic reaction was stopped with 0.25 mL of acetic acid (30%), and the absorbance was read at 410 nm. The trypsin-like activity was reported as activity units (Abs 410/min)/mg protein [22].

**Amylase Activity**

Soluble corn starch was used as substrate. Five *μ*L of the enzymatic extract, 500 *μ*L Tris-HCl buffer (50 mM, pH 7.5), and 500 *μ*L of soluble starch (1%) were mixed. After 10 min of incubation, 200 *μ*L of 2N sodium carbonate, and 1.5 mL of dinitrosalicylic acid (DNS) were added; the mixture was agitated and warmed to boil in water bath for 15 min, thereafter 7.3 mL of distilled water were added. Absorbance was read at 550 nm. Amylase units were reported as Units (Abs 550/min)/mg protein [23].

**Lipase Activity**

*β*-naphthyl caprylate was used as substrate. Ten *μ*L of the enzymatic extract, 100 *μ*L of sodium tauracholate 100 mM, 1900 *μ*L Tris-HCl buffer 50 mM, pH 7.2, and 20 *μ*L *β*-naphthyl caprylate 200 mM were mixed and incubated at room temperature. After 30 min, a solution composed by 20 *μ*L Fast Blue BB 100 mM, 200 *μ*L trichloroacetic acid 0.72 N, and 2.71 mL ethanol : ethyl acetate (1 : 1) were added. Absorbance was read at 540 nm. Lipase units were reported as Units (Abs 540/min)/mg protein [24].

At the end of the trial, samples of bioflocs from AS and HS were collected by filtering 2 L of water in a nylon sieve (0.5 mm mesh). The chemical proximate composition of the bioflocs was analyzed to determine protein and lipid contents. The protein content was measured following the methodology described by Lowry et al. [25] with modifications [26]; the carbohydrate concentration was estimated by the “phenol-sulfuric acid” method [27]. Total lipids were extracted according to Bligh and Dyer [28], and the quantification was done following the methodology described by Pande et al. [29]. Ash was estimated by incineration at 550°C.

Regarding statistical analyses, water quality data were studied by a repeated measure analysis of variance (ANOVA). Production parameters (except survival) and enzymatic activity were analyzed by one-way ANOVA, and statistical differences were identified by a post hoc Tukey test. Survival data were analyzed by a chi-square test.

## 3. Results

No significant differences in temperature, salinity, or dissolved oxygen were found among treatments. pH was significantly lower in the HS compared to AS and the control. The chlorophyll-a concentrations were extremely higher in the AS and the control (>160 mg*·*m^3^) compared to the HS (Table 1).

Regarding production response of shrimp, some significant differences were observed among treatments (Table 2). The weight gain was significantly higher in the control (1.58 g) compared to the HS (0.87 g) and AS (1.02 g), which were not significantly different. A similar response was observed for specific growth rate (SGR), but no significant differences were detected. Contrarily, the survival was better in the HS and AS (≥68%) compared to the control (<40%). Final biomass also resulted to be significantly greater in the AS and HS when compared to the control. The FCRs were low in all treatments (<1), but the values registered in HS and AS were significantly lower than the control. 

With respect to enzymatic activities, no significant differences were observed among treatments for lipase activity (0.002-0.003 Abs/min/mg protein) (Table 3). Trypsin-like activity was slightly higher in the HS, but without significant differences respect to AS and control; however, the amylase activity resulted to be greater in the HS compared to AS and control. The chemical proximate analysis of the bioflocs (Table 4) revealed higher protein content in the HS, whereas the lipid content was greater in bioflocs from the AS. Similar ash and carbohydrate contents were found in both biofloc types.

Regarding the biotic communities generated within the experimental units, greater concentrations of diatoms and cyanobacteria were registered in the water of AS and control; whereas higher concentrations of heterotrophic bacteria were observed in the HS (Table 5). Regarding microorganisms attached to substrates, the biofilm of AS was mainly composed by diatoms and cyanobacteria, whereas heterotrophic bacteria and filamentous bacteria were dominant in the HS (Table 5).

## 4. Discussion

All the environmental variables during the trial were within the range considered suitable for the culture of white shrimp [30]. The pH was lower in the heterotrophic system compared to the control, suggesting a reducing condition in such treatment, probably due to the activity of heterotrophic bacteria, which release CO_2_ to the water column causing a pH decrease. Contrarily, in the control and AS, where the photosynthesis was enhanced, the phytoplankton produced CO_2_ during the night, but sequesters it during the day, causing pH increases. Low pH levels ranging from 6.4 to 7.7 in the culture of *F. paulensis* have been reported when using microbial flocs [11].

The analysis of the biotic communities generated into the experimental units suggests the success of the strategy used. Heterotrophic and autotrophic communities were generated by the addition of the adequate nutrients and manipulation the environmental conditions. As expected, the concentration of chlorophyll-a was much greater in the autotrophic system and the control compared to the heterotrophic. The manipulation of inorganic nutrients, combined with the direct exposure of sunlight in AS and C, was the cause of the great differences. The levels of chlorophyll-a in AS and C, were always much higher than those usually reported for semi-intensive aquaculture ponds [8]. However, similar chlorophyll concentrations other than those we found, have been achieved by performing similar modifications [11].

The productive response of shrimp was different among the experimental treatments and the control. The best growth observed in the control was mostly a consequence of the lower survival, which implied a lower shrimp density in those units. An inverse correlation between density and survival is commonly observed [30]. The best survival (almost 70%) recorded in the autotrophic and heterotrophic systems, is very acceptable for an intensive pregrowth [31] considering that mass mortalities could occur at larvae and postlarvae phases. For instance, survivals as low as 19% were recorded in penaeid shrimp reared at pregrowout phase and using formulated feed exclusively; however, the survival was improved to 26% when used bioflocs exclusively and to 48% when combined formulated feed and bioflocs [10]. Moreover, survivals around 90% have been documented using bioflocs [11].

The feed conversion ratios recorded in the AS, HS, and inclusively in the control were lower than those commonly reported in commercial farms and strongly suggest that shrimp use the bioflocs and biofilms as an important source of nutrition. It has been argued that the combination of artificial food with natural food in form of biofloc and biofilm may complete the nutritional requirements of cultured organisms, improving their growth and nutritional performance [16]. The conditions of zero water exchange probably also contributed to the decrease of the FCR in all the treatments because there was not any release of nutrients in effluents, which favored the formation of a nutrient cycling through the food chain. Nutrient cycling has been documented in systems without water exchange in which natural feed was promoted [32–34].

The slight higher activity of trypsin observed in the HS could be related to the higher concentration of protein found in the bioflocs sampled from such treatment. The amylase activity was also higher in the HS, suggesting a higher digestive activity in shrimp fed on heterotrophic bacteria. Significant increases of digestive enzyme activities of *P. vannamei* larvae have been observed when the bacterium *Bacillus coagulans* is used as probiotic [19]. In addition, some species of bacteria are able to produce exogenous enzymes [35], but the contribution of exogenous enzymes themselves to the digestion process of fish and crustacean seems to be insignificant; however, the presence of bacteria with their respective exogenous enzymes could stimulate the production of endogenous enzymes by fish and shrimp [19, 20, 36]. In the present study, the slight increase of trypsin activity, and the greater amylase activity could be explained by the presence of heterotrophic bacteria in the HS. 

The higher protein concentration in bioflocs of the HS may be related to the chemical composition of heterotrophic bacteria and other organisms associated to bioflocs and biofilms. Authors have considered microorganisms as contributors to the nutrition of *F. paulensis*, in terms of proteins and lipids [17]. The higher concentration of lipids in the autotrophic system are probably due to the great density of microalgae, which are recognized as an important source of these macronutrients [37, 38].

In general, the results obtained suggest feasibility of rearing *L. vannamei *from early larvae phases using both, heterotrophic or autotrophic-based systems without water exchange, and perhaps most importantly, without the use of Artemia which is an expensive and yet considered an essential supply in shrimp aquaculture. In addition, the zero water exchange condition assures the biosecurity of the system. Finally, it is important to perform studies to evaluate the effect of the diverse bioflocs and biofilms on the postharvest quality and organoleptic characteristics of shrimp.

## Figures and Tables

**Table 1:** Means ± SD of the environmental variables during the pregrowth of *L. vannamei* in the autotrophic (AS), heterotrophic (H), and the control systems.

	Temperature (°C)	Salinity (PSU)	DO (mg*·*L^−1^)	pH	Chlorophyll-a (mg*·*m^−3^)
C	31.7 ± 1.13^a^	37.3 ± 0.57^a^	6.2 ± 3.7^a^	7.8 ± 0.2^a^	166.9 ± 119.5^a^
AS	31.7 ± 1.19^a^	37.5 ± 0.67^a^	6.5 ± 4.2^a^	7.7 ± 0.2^a^	261.9 ± 158.2^a^
HS	30.5 ± 0.65^a^	36.3 ± 0.77^a^	6.5 ± 3.1^a^	7.5 ± 0.13^b^	5.5 ± 3.6^b^

Different letters in the same column indicate significant differences (*P* < 0.05).

**Table 2:** Means ± SD of the production parameters of *L. vannamei* in the autotrophic (AS), heterotrophic (H), and the control systems.

	Survival (%)	Weight gain (g)	Total biomass (g*·*m^−3^)	FCR	SGR%*·*week^−1^
C	39.6 ± 2.87^b^	1.58 ± 0.31^a^	417.6 ± 16.5^b^	0.61 ± 0.04^a^	51.8 ± 7.7^a^
AS	68.0 ± 3.42^a^	0.87 ± 0.31^b^	475.3 ± 22.9^a^	0.54 ± 0.03^b^	45.0 ± 6.6^a^
HS	68.4 ± 2.31^a^	1.02 ± 0.35^b^	449.4 ± 18.8^a^	0.55 ± 0.04^b^	46.9 ± 8.4^a^

Different letters in the same column indicate significant differences (*P* < 0.05).

**Table 3:** Means ± SD of digestive enzymes activity of *L. vannamei* in the autotrophic (A), heterotrophic (H), and control systems.

	Trypsin (abs/min/mg protein)	Amylase (abs/min/mg protein)	Lipase (abs/min/mg protein)
C	0.24 ± 0.03^a^	0.21 ± 0.02^a^	0.003 ± 0.001^a^
AS	0.25 ± 0.04^a^	0.23 ± 0.05^a^	0.003 ± 0.001^a^
HS	0.28 ± 0.07^a^	0.28 ± 0.06^b^	0.002 ± 0.001^a^

Different letters in the same column indicate significant differences (*P* < 0.05).

**Table 4:** Chemical proximate composition in dry basis of bioflocs collected from the autotrophic (AS) and heterotrophic systems (HS).

Percentage	HS	AS
Protein	17.5	11.5
Lipid	6.5	13.3
Carbohydrate	34.9	35.4
Ash	41.1	39.8

**Table 5:** Biotic communities registered within the different treatments.

	AS	HS	Control
	Microalgae communities in water (cells*·*mL^−1^ **)**		
Cyanobacteria (×10^6^)	3.44 ± 0.18^b^	0.01 ± 0.01^a^	3.33 ± 0.21^b^
Diatoms (×10^4^)	11.8 ± 1.3^b^	—	10.6 ± 1.2^b^
Heterotrophic bacteria (×10^5^)	<0.001^a^	1.81 ± 0.28^b^	<0.001^a^
	Microalgae and bacterial communities in artificial substrates (cells*·*cm^−2^)		
Diatoms and cyanobacteria (×10^4^)	2.2 ± 0.3	—	—
Heterotrophic and filamentous cyanobacteria (×10^5^)	—	2.6 ± 0.4	—

Different letters in the same row indicate significant differences (*P* < 0.05).

## References

[1] Tacon AGJ (2002). Thematic review of feed and feed management practices in shrimp aquaculture.

[2] Martínez-Córdova LR, Porchas MM, Cortés-Jacinto E (2009). Shrimp mexican and world: sustainable activity or polluting industries?. *Revista Internacional de Contaminacion Ambiental*.

[3] Anderson RK, Parker PL, Lawrence AA (1987). A 13C/12C tracer study of the utilization of presented feed by a commercially important shrimp *Penaeus vannamei* in a pond growth out system. *Journal of the World Aquaculture Society*.

[4] Martínez-Córdova LR, Campaña-Torres A, Porchas-Cornejo MA (2002). Promotion and contribution of biota in low water exchange ponds farming blue shrimp *Litopenaeus stylirostris* (Stimpson). *Aquaculture Research*.

[5] Martínez-Córdova LR, Peña-Messina E (2005). Biotic communities and feeding habits of *Litopenaeus vannamei* (Boone 1931) and *Litopenaeus stylirostris* (Stimpson 1974) in monoculture and polyculture semi-intensive ponds. *Aquaculture Research*.

[6] Campaña-Torres A, Martínez-Córdova LR, Villarreal-Colmenares H, Hernández-López J, Ezquerra-Brauer JM, Cortés-Jacinto E (2009). Effect of the rotifer *Brachionus rotundiformis* (Tschugunoff, 1921) addition on water quality and production, in super-intensive cultures of the Pacific white shrimp *Litopenaeus vannamei* (Boone, 1931). *Revista de Biologia Marina y Oceanografia*.

[7] Muangkeow B, Ikejima K, Powtongsook S, Gallardo W (2011). Growth and nutrient conversion of white shrimp *Litopenaeus vannamei* (Boone) and Nile tilapia *Oreochromis niloticus* L. in an integrated closed recirculating system. *Aquaculture Research*.

[8] Porchas-Cornejo MA, Martínez-Córdova LR, Ramos-Trujillo L, Hernández-López J, Martínez-Porchas M, Mendoza-Cano F (2011). Effect of promoted natural feed on the production, nutritional, and immunological parameters of *Litopenaeus vannamei* (Boone, 1931) semi-intensively farmed. *Aquaculture Nutrition*.

[9] Farzanfar A (2006). The use of probiotics in shrimp aquaculture. *FEMS Immunology and Medical Microbiology*.

[10] Emerenciano M, Ballester ELC, Cavalli RO, Wasielesky W (2011). Effect of biofloc technology (BFT) on the early postlarval stage of pink shrimp *Farfantepenaeus paulensis*: growth performance, floc composition and salinity stress tolerance. *Aquaculture International*.

[11] Ballester ELC, Abreu PC, Cavalli RO, Emerenciano M, de Abreu L, Wasielesky W (2010). Effect of practical diets with different protein levels on the performance of *Farfantepenaeus paulensis* juveniles nursed in a zero exchange suspended microbial flocs intensive system. *Aquaculture Nutrition*.

[12] Becerra-Dórame MJ, Martínez-Córdova LR, Martínez-Porchas M, López-Elías JA (2011). Evaluation of autotrophic and heterotrophic microcosm-based systems on the production response of *Litopenaeus vannamei* intensively nursed without Artemia and with zero water exchange. *Israeli Journal of Aquaculture-Bamidgeh*.

[13] Audelo-Naranjo JM, Martínez-Córdova LR, Voltolina D (2010). Nitrogen budget in intensive cultures of *Litopenaeus vannamei* in mesocosms, with zero water exchange and artificial substrates. *Revista de Biología Marina y Oceanografía*.

[14] Audelo-Naranjo JM, Martínez-Córdova LR, Voltolina D, Gómez-Jiménez S (2011). Water quality, production parameters and nutritional condition of *Litopenaeus vannamei* (Boone, 1931) grown intensively in zero water exchange mesocosms with artificial substrates. *Aquaculture Research*.

[15] Abreu PC, Ballester ELC, Odebrecht C (2007). Importance of biofilm as food source for shrimp (*Farfantepenaeus paulensis*) evaluated by stable isotopes (*δ*13C and *δ*15N). *Journal of Experimental Marine Biology and Ecology*.

[16] Avnimelech Y (2009). *Biofloc Technology, a Practical Guide Book*.

[17] Fernandes Da Silva C, Ballester E, Monserrat J, Geracitano L, Wasielesky W, Abreu PC (2008). Contribution of microorganisms to the biofilm nutritional quality: protein and lipid contents. *Aquaculture Nutrition*.

[18] D’Abramo LR, Conklin DE, Akiyama DM (1997). *Crustacean Nutrition. Advances in World Aquaculture*.

[19] Zhou XX, Wang YB, Li WF (2009). Effect of probiotic on larvae shrimp (*Penaeus vannamei*) based on water quality, survival rate and digestive enzyme activities. *Aquaculture*.

[20] Ziaei-Nejad S, Rezaei MH, Takami GA, Lovett DL, Mirvaghefi AR, Shakouri M (2006). The effect of *Bacillus* spp. bacteria used as probiotics on digestive enzyme activity, survival and growth in the Indian white shrimp *Fenneropenaeus indicus*. *Aquaculture*.

[21] Burford MA, Sellars MJ, Arnold SJ, Keys SJ, Crocos PJ, Preston NP (2004). Contribution of the natural biota associated with substrates to the nutritional requirements of the post-larval shrimp, *Penaeus esculentus* (Haswell), in high-density rearing systems. *Aquaculture Research*.

[22] Erlanger BF, Kokowsky N, Cohen W (1961). The preparation and properties of two new chromogenic substrates of trypsin. *Archives of Biochemistry and Biophysics*.

[23] Vega-Villasante F, Nolasco H, Civera R (1993). The digestive enzymes of the Pacific brown shrimp *Penaeus californiensis*. I.—properties of amylase activity in the digestive tract. *Comparative Biochemistry and Physiology*.

[24] Versaw WK, Cuppett SL, Winters DD, Williams LE (1989). An improved colorimetric assay for bacterial lipase in nonfat dry milk. *Journal of Food Science*.

[25] Lowry OH, Rosenbrough NJ, Farr AL, Randall RJ (1951). Protein measurement with the Folin phenol reagent. *The Journal of Biological Chemistry*.

[26] López-Elías JA, Voltolina D (1993). Semicontinuos cultures of four microalgae with a nonconventional medium. *Ciencias Marinas*.

[27] Dubois M, Gilles KA, Hamilton JK, Rebers PA, Smith F (1956). Colorimetric method for determination of sugars and related substances. *Analytical Chemistry*.

[28] Bligh EG, Dyer WJ (1959). A rapid method of total lipid extractionand purification. *Canadian Journal of Biochemistry and Physiology*.

[29] Pande SV, Khan RP, Venkitasubramanian TA (1963). Microdetermination of lipids and serum total fatty acids. *Analytical Biochemistry*.

[30] Martínez Córdova LR (2009). *Camaronicultura Sustentable*.

[31] Campaña-Torres A, Martínez-Córdova LR, Villarreal-Colmenares H, Cortés-Jacinto E (2010). Evaluation of different concentrations of adult live Artemia (*Artemia franciscana*, Kellogs 1906) as natural exogenous feed on the water quality and production parameters of *Litopenaeus vannamei* (Boone 1931) pre-grown intensively. *Aquaculture Research*.

[32] Avinmelech Y, Verdegem MCJ, Kurup M, Keshavanath P (2008). Sustainable land-based aquaculture: rational utilization of water, land and feed resources. *Mediterranean Aquaculture Journal*.

[33] Crispim MC, Vieira ACB, Coelho SFM, Medeiros AMA (2007). Nutrient uptake efficiency by macrophyte and biofilm: practical strategies for small-scale fish farming. *Acta Limnologica Brasiliensis*.

[34] Jirsa DO, Davis DA, Arnold CR (1997). Effects of dietary nutrient density on water quality and growth of red drum *Sciaenops ocellatus* in closed systems. *Journal of the World Aquaculture Society*.

[35] Moriarty DJW (1998). Control of luminous *Vibrio* species in penaeid aquaculture ponds. *Aquaculture*.

[36] Kurokawa T, Shiraishi M, Suzuki T (1998). Quantification of exogenous protease derived from zooplankton in the intestine of Japanese sardine (*Sardinops melanotictus*) larvae. *Aquaculture*.

[37] Rodolfi L, Bassi N, Parente I Lipid production from marine microalgae: strain selection, induction of lipid synthesis and outdoor cultivation in pilot photobioreactors.

[38] Rodolfi L, Zittelli GC, Bassi N (2009). Microalgae for oil: strain selection, induction of lipid synthesis and outdoor mass cultivation in a low-cost photobioreactor. *Biotechnology and Bioengineering*.

